# Technology, Yoga, and Hallway Walks to Maintain Function and Self-Efficacy During the Home-Bound Months of Winter

**DOI:** 10.1093/geroni/igaa057.3094

**Published:** 2020-12-16

**Authors:** Kim LeBard-Rankila

**Affiliations:** University of Wisconsin Superior, Superior, Wisconsin, United States

## Abstract

Winter months present individuals with a unique challenge, decreased daily movement that can lead to decreased physical and emotional health. Snow and ice cause hazardous walkways and driving conditions that can lead to falls and decreased travel out of the home. For those that live in states that have four seasons, when spring comes many of them have spent a large majority of their daily lives indoors for 3-5 months. This isolation can cause decreased daily movement, aggravate medical conditions, challenge healthy eating habits, and cause social isolation. Participants for this study live in a 32-apartment building, which all occupants are independent with daily living skills and are 60 years or older. The study assessed if motivational texting, yoga in the apartment’s community center, and short hall-way walks can help maintain or increase self-efficacy. Flexibility, hand-grip strength, and perceived quality of life were assessed.

